# Trans-arterial embolization of acquired uterine arteriovenous malformation after Cesarean section: A case series

**DOI:** 10.18502/ijrm.v17i2.3991

**Published:** 2019-03-20

**Authors:** Achmad Kemal Harzif, OG (REI), Agrifa Haloho, Melisa Silvia, Gita Pratama, OG(REI), MRepSc, Yuditiya Purwosunu, OG(MFM), Ph.D., Aria Wibawa, OG(MFM), Prijo Sidipratomo, Rad(IR), Ph.D., Jacub Pandelaki, Rad(IR), Ph.D.

**Affiliations:** ^1^Division of Immuno-Endocrinology and Fertility, Department of Obstetrics and Gynecology, Faculty of Medicine, Universitas Indonesia, Dr. Cipto Mangunkusumo Hospital Jakarta, Indonesia.; ^2^Department of Obstetrics and Gynecology, Faculty of Medicine, Universitas Indonesia, Dr. Cipto Mangunkusumo Hospital Jakarta, Indonesia.; ^3^Indonesian Reproductive Medicine Research and Training Center, Department of Obstetrics and Gynecology, Faculty of Medicine, Universitas Indonesia, Dr. Cipto Mangunkusumo Hospital Jakarta, Indonesia.; ^4^Division of Maternal-Fetal Medicine, Department of Obstetrics and Gynecology, Faculty of Medicine, Universitas Indonesia, Dr. Cipto Mangunkusumo Hospital Jakarta, Indonesia.; ^5^Department of Radiology, Faculty of Medicine, Universitas Indonesia, Dr. Cipto Mangunkusumo Hospital Jakarta, Indonesia.

**Keywords:** *Trans-arterial embolization*, *Acquired uterine arteriovenous malformation*, *Cesarean section*

## Abstract

**Background:**

Acquired uterine arteriovenous malformation (AVM) is a rare condition due to traumatic episodes in cesarean section. The patient can suffer from life-threatening hemorrhage or recurrent vaginal bleeding. Establishing this diagnosis is difficult, often misdiagnosed due to lack of information and number of cases. Trans-Arterial Embolization (TAE) procedure is rarely performed in our center. All of the cases were found with history of massive bleeding and diagnosed lately after recurrent bleeding history. Even though promising, one of our cases failed to be managed with TAE. It is important to diagnose early symptoms of AVM in order to prevent the life threatening event.

**Case presentation:**

In these case series, four cases of AVMs after cesarean procedures will be reviewed. One could be diagnosed in less than a month but the other three took several months. The symptom of vaginal bleeding might occur a few weeks after the procedure is done, and most patients need transfusion and hospitalization. Three out of four patients were initially sent to the hospital in order to recover from shock condition, and one patient was sent for a diagnostic procedure. AVMs diagnostic was established with ultrasound with or without angiography. Three of our cases were succeeded by performing TAE procedure without further severe vaginal bleeding. One case failed to be treated with embolization and had to proceed with hysterectomy.

**Conclusion:**

AVM should be considered early-on in patient with abnormal uterine bleeding and history of cesarean section. Embolization is still the first-choice treatment of AVMs, otherwise definitive treatment is hysterectomy in a patient without fertility need, or impossible to perform TAE.

## 1. Introduction 

Arteriovenous malformation (AVM) is a rare condition complicating procedures such as cesarean section, hysteroscopy, or curettage. Cesarean section places second in leading clinical history associated with AVMs (1). The incidence and prevalence is difficult to determine, only some respond to medical treatment and several remain undiagnosed (2). The patient can suffer from life-threatening hemorrhage or recurrent vaginal bleeding. Misdiagnosis is commonly found due to lack of information and a few numbers of cases. The choice of management of this patient varies from hormonal treatment, open surgical, or minimally invasive such TAE (1). In this report, we present our experience in managing four cases of AVMs, including the undiagnosed disease, life threatening consequences, and the treatment.

## 2. Case Presentation

### Case 1

**Figure 1:**
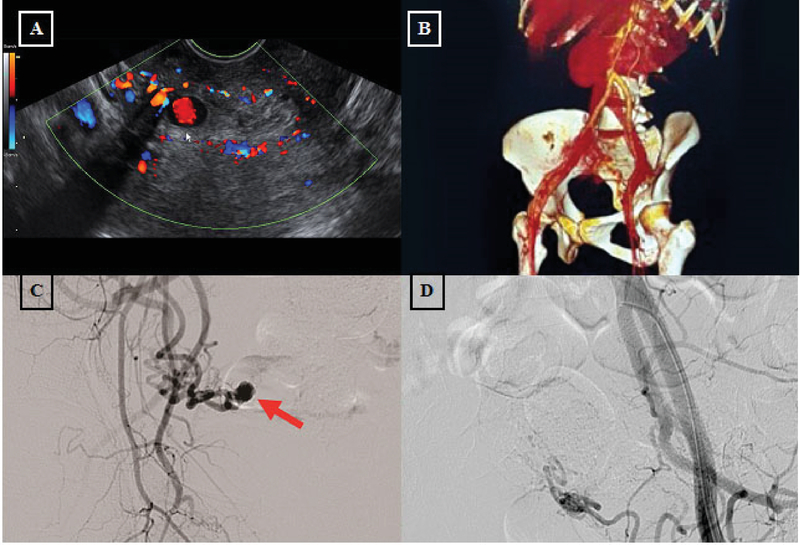
**(A)** Doppler study showed hematoma connected with right uterine artery; (**B**) Hyper vascular pattern originated from right uterine artery; (**C) **Aneurism originated from right uterine artery pre-TAE; and (**D)** Post micro-coil insertion.

A 35–yr-old woman, parity 3, came with recurrent vaginal bleeding with a history of cesarean section due to preeclampsia 25 days ago. She had a history of two-time cesarean section due to hypertension in pregnancy. She used pills and progestin injection for controlling the pregnancy. She didn't smoke and no one in her family had the same problem. She experienced two times hypovolemic shock and hemoglobin level was 3.9 g/dl. She had packed red cell (PRC) transfusion and was able to reach hemoglobin level 10.5 g/dl. In the first episode of shock, the gynecologist didn't find abnormality. Suspicion of AVM was established in the second examination. She was referred to the tertiary hospital for further examination. Right hematoma of 13×33 mm was found connected with right uterine artery on the ultrasound examination corresponding to AVM (Figure 1A). The patient was then referred to vascular surgery department where she underwent Multi-Slice Computed Tomography (MSCT) angiography, and hyper vascular pattern was discovered from the right uterine artery (Figure 1B). Embolization was conducted after stabilization. Guiding wire was inserted from femoral artery to external iliac artery, common iliac artery, and aorta. Digital subtraction angiography (DSA) was systematically performed from the aorta to the iliac artery. Aneurysm was discovered originating from right uterine artery (Figure 1C). Polyvinyl Alcohol (PVA) embolization was then performed and gel-foam with coil. Micro-catheter was inserted selectively to one of the uterine artery branch until it reached the aneurysm followed by coil insertion. Diagnostic catheter was then placed at the internal iliac artery and contrast was administered thereafter, no aneurism nor other malformation was found (Figure 1D).

Five days after TAE, she had another vaginal bleeding and she underwent hysterectomy.

### Case 2

**Figure 2:**
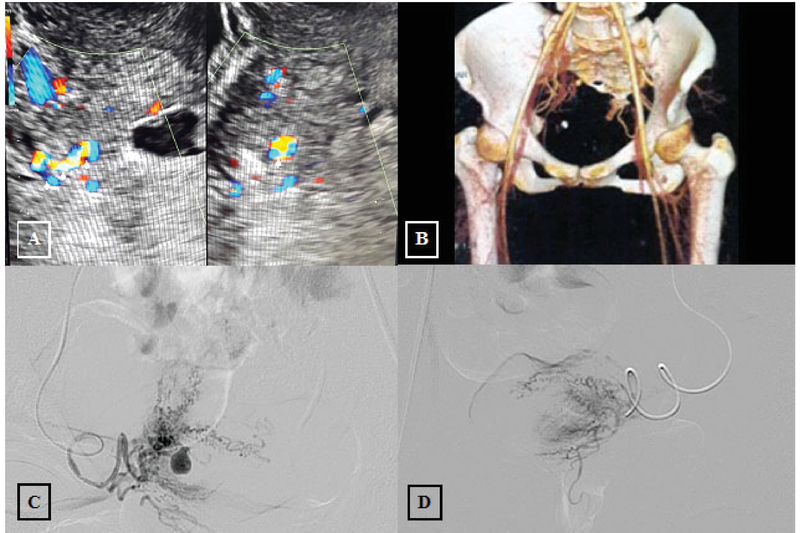
**(A) **New vascularization on the cesarean scar with vessel enlargement; (**B)** Lobulated oval lesion on CT Angiography; (**C) **Aneurism at the right ascending uterine artery; and (**D) **TAE procedure found that there were no aneurism and hypervascularization.

A 27-year-old woman came with profuse vaginal bleeding with a history of cesarean section 60 days before admission due to non-reassuring fetal heart pattern. She didn't use any contraception. She had no metabolic disease and never took any routine medication. She didn't smoke and no one in her family had the same complaint. She started to experience bleeding a week after the procedure was done. Some episodes were just spotting but some episodes were more like menstrual blood. Once she got profuse vaginal bleeding and blood transfusion with hemoglobin level (6 gr/dl). She was planned for tertiary hospital referral. Unfortunately, another attack of bleeding happened that also required hospitalization and blood transfusion. From Doppler study, new vascularization was seen on the right area of cesarean scar with vessel enlargement corresponding to aneurysm or vascular malformation (Figure 2A). From the CT Angiograph, new lobulated oval lesion was visible after the administration of contrast sized 0.67×0.47×0.75 cm, originating from distal branch of right uterine artery corresponding to right uterine artery aneurysm (Figure 2B). Catheter was placed in the right iliac artery and aneurism was proved originating from the ascending branch of right uterine artery (Figure 2C). By using micro-catheter, selective right uterine artery catheterization was performed. Aneurysm was surrounded by hypervascularization derived from arterial branches. Embolization was performed using PVA 710–1000 microns. There was no aneurysm nor hypervascularization was seen after the procedure (Figure 2D). She had no vaginal bleeding history after procedure.

### Case 3

**Figure 3:**
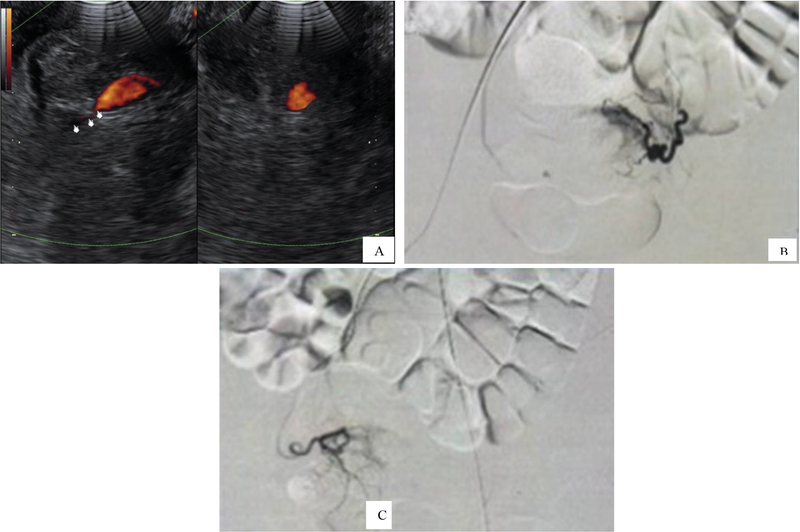
**(A) **Hematoma at left adnexa connected to left uterine artery; **(B) **Saccular dilatation of left uterine artery with sign of extravasation of the artery.** (C)** Embolization on left uterine artery using PVA and gel-foam.

An 18-year-old woman came to hospital with chief complain of vaginal bleeding with a history of cesarean section 49 days before admission due to premature rupture of membrane. She never took any medication for systemic disease and never had the same vaginal bleeding before pregnancy. She wasn't a smoker and no one in her family had the same complaint. She didn't take any birth-controlling pills or injection. The first vaginal bleeding happened a week after cesarean section without any abnormality being found. Recurrent bleeding happened and PRC transfusion was administered due to severe anemic condition with the lowest hemoglobin being 4 gr/dl a week later. Another episode of vaginal bleeding resulted in lowering hemoglobin to 7.72 g/dl and caused an emergency referral to our center. From transvaginal ultrasound examination, at the left area of cesarean scar, hematoma was seen in a non-homogenous area. It was connected to the left uterine artery branch, then diagnosed as AVMs and planned for TAE (Figure 3A). Sheath 5F on left common femoral artery was introduced. The end of catheter was placed on distal left internal iliac artery. Saccular dilatation of left uterine artery was found with sign of extravasation of the artery, and also hypervascularization and hypertrophy of left uterine artery (Figure 3B). Embolization was performed on the left uterine artery using PVA and gel-foam. There was no aneurysm on the post embolization (Figure 3 C).

### Case 4

**Figure 4:**
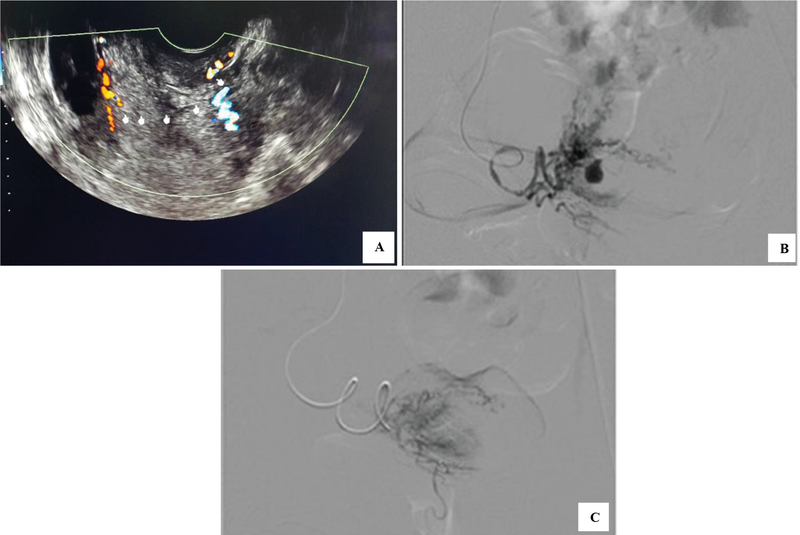
**(A)** AVM at left cesarean scar.** (B)** AVM seen at the branch of left uterine artery continued with the selective catheterization.** (C**) TAE procedure found no AVMs.

A 32-year-old woman, parity 2 post-cesarean section 4 months ago due to placenta previa came with hypovolemic shock due to abnormal uterine bleeding. She had no experience of vaginal bleeding before or during pregnancy. The first pregnancy was spontaneous delivery. She took hormonal pills for birth controlling. She didn't smoke and no one in her family had the same complain. She had two previous vaginal bleeding with unremarkable gynecology and ultrasound examination. Hemoglobin level was 4.2 g/dl. She was referred to tertiary hospital for further evaluation. There was AVM at the area of left cesarean scar (Figure 4A) and hence planned for TAE. The catheter was inserted at the left iliac artery. It was seen that the right uterine artery was normal and found AVM at the branch of left uterine artery (Figure 4B) continued with the selective catheterization. There was no AVMs after the procedure nor a history of bleeding on policlinic follow-up (Figure 4C). Summary of four cases can be seen in Table I.

**Table 1:** Clinical summary of four cases of AVMs after caesarean section treated with TAE.


	Case 1	Case 2	Case 3	Case 4
Age (yr)	35	27	18	32
Parity	P3	P1	P1	P2
History of Caesarean Section	Preeclampsia	Non reassuring Fetal status	PPROM	Placenta Previa
Days of Diagnostic After Caesarean Section	25	60	49	131
AVM Side	Right	Right	Left	Left
Initial Hemoglobin (g/dl)	3.9	7.6	7.72	4.2
History of Transfusion	Yes	Yes	Yes	Yes
Outcome	Recurrent bleeding 30 days after TAE and underwent hysterectomy	No history of Abnormal Uterine Bleeding	No history of Abnormal Uterine Bleeding	No history of Abnormal Uterine Bleeding
Note: AVM: Arteriovenous Malformation; P: Parity; and PPROM: Preterm Premature Rupture of Membranes.				

## 3. Discussion

AVM is a rare condition about 1–2% as complication of postpartum and consequence of increased cesarean deliveries, with symptom of vaginal or intra-peritoneal bleeding (3, 4). The diagnosis is difficult, often misdiagnosis with retained placenta. The cause of acquired AVMs is traumatic episode of uterine artery during cesarean section or myomectomy. Sometimes, infection contributes to the development of AVM (5). Also, the AVMs were reported as the results of cesarean scar pregnancy, conservative accrete treatment, and laparoscopic instrumentation of the uterine (6–8). There was no specific technique or cesarean indication that caused the AVMs directly in literature findings, also in our case the indications in four cases varies.

The patient usually comes with postpartum hemorrhage after cesarean section. In this report, we learned that initially all of the cases were diagnosed incorrectly. The episode was commonly acute and found in hypovolemic shock that may bring the patient in life-threatening condition and increased rate of transfusion. The vaginal bleeding symptom came few weeks after the procedures and most of the patients need transfusion and being hospitalized. Three of four in our cases were sent after stabilization in other hospital with a history of shock condition and sent for diagnostic procedure. Some of them also got hormonal treatment in some cases for abnormal bleeding management and reported lighter menstrual bleeding (5).

Because clinical finding included only bleeding, the ultrasound and angiography have important role in diagnostic AVMs (3, 9). The gray-scale ultrasound examination is common but unable to conclude any AVM. Our patient was already examined before and the physician didnot find any pathology. The doppler study is the main screening tool in AVM. The ultrasound result of AVM is not specific for retained placenta or AVM but a hypo-echoic cystic or tubuliform area within myometrium and bidirectional high-velocity- and low-resistance blood flow increased suspicion of AVM and needed CT angiography as a gold standard (4, 10–12). MRI is able to diagnose the AVMs with voluminous uterus, ill define mass, and focal or diffuse interruption zone (12). CT angiography can show if there is any extension to the extra uterine or involvement or feeding artery, so that every vascular malformation can be seen (13).

An asymptomatic AVM can be treated with expectant management. When there are repeated symptoms, the traditional management, hysterectomy, or artery ligation can be chosen (14, 15). Some case reports already proposed hormonal treatment in managing the AVMs. The origin of the AVMs in pregnancy may correlate with estrogen, vascular endothelial growth hormone, or another hormone in pregnancy. Based on that hypothesis, the gonadotrophin agonist was given by the down-regulated estrogen mechanism (16). Gonadotropin-releasing hormone was reported reducing the size of AVM (17). Danazol and progestin can decrease the bleeding (18). The intrauterine hormonal device is also able to decrease the amount of blood loss, but the expulsion of device is also reported (5). In our cases, we preferred using surgery technique TAE due to the patients' history of acute bleeding, and the first choice in managing AVM was selective embolization.

Three of our cases were succeeded with TAE procedure without any severe vaginal bleeding reported. One patient got another bleeding after TAE procedure. The success rate of TAE in resolution of the lesion is about 85%. The others needed another surgical procedures, repeated TAE, laparoscopic approach, or hysterectomy (1). Not only as a definitive treatment, but also hysterectomy is a choice when the facility of TAE is absent and for the patient who doesn't desire fertility. Rarely, clinical events after TAE are amenorrhea and menopausal symptom. In general, TAE procedures not specifically for AVMs found there was no differences of ovarian function (19).

We only had four cases in five years' period. This shows that early diagnostic and treatment of AVM are far from ideal. Also the diagnostic procedures were different from each other. We are very far from making a standardized management because of the limitations of the case and facilities. But reporting this, we move forward and encourage to preserve uterine function by TAE in developing countries.

As a conclusion, artery venous malformation is a consequences of increased cesarean delivery. The diagnostic isn't always easy but has to be considered in patient with history of cesarean section. Embolization is still the first choice of AVMs, otherwise definitive treatment is hysterectomy in patient without fertility needs or it is impossible to perform TAE.

##  Conflict of Interest

The authors have no conflict of interest.
